# Safety and efficacy of rituximab-free ABO incompatible kidney transplantation: a German multicenter cohort study

**DOI:** 10.3389/fneph.2026.1899385

**Published:** 2026-07-17

**Authors:** Ulrich Pein, Klemens Budde, Lutz Liefeldt, Anja Gäckler, Rolf Weimer, Hristos Karakizlis, Claudia Sommerer, Louise Benning, Christine Kurschat, Dirk Stippel, Ana Harth, Julia Weinmann-Menke, Martina Koch, Birgit Kortus-Goetze, Stefan Reuter, Nils Lachmann, Anja Mühlfeld

**Affiliations:** 1 Martin-Luther-University Halle-Wittenberg, University Hospital Halle (Saale), Department of Nephrology, Halle, Germany; 2 Department of Nephrology, Charite - Universitatsmedizin Berlin, Berlin, Germany; 3 Department of Infectious Diseases and Nephrology, University Hospital Essen, Essen, Germany; 4 University of Giessen, Department of Internal Medicine, Giessen, Germany; 5 University Hospital Heidelberg, Department of Nephrology, Heidelberg, Germany; 6 Department II of Internal Medicine and Center for Molecular Medicine Cologne, University of Cologne, Faculty of Medicine and University Hospital Cologne, Cologne, Germany; 7 Department of General, Visceral, Cancer and Transplant Surgery, University Hospital Cologne, Cologne, Germany; 8 Department of Nephrology, Hospital Köln-Merheim, Cologne, Germany; 9 Department of Nephrology, University Hospital Mainz, Mainz, Germany; 10 Department of General-, Visceral- and Transplantation Surgery, University Hospital Mainz, Mainz, Germany; 11 Department of Nephrology, University Hospital Marburg, Marburg, Germany; 12 Department of Medicine D, University Clinics Münster, Münster, Germany; 13 Institute for Transfusion Medicine, Histocompatibility and Immunogenetics Laboratory, Charité Berlin, Berlin, Germany; 14 Department of Nephrology, Uniklinik RWTH Aachen, Aachen, Germany

**Keywords:** ABO incompatible, infectious complications, living kidney transplantation, outcome, rituximab

## Abstract

**Background:**

ABO-incompatible living kidney transplantation (ABOi-LKT) has become an established procedure with long-term patient and graft survival comparable to ABO-compatible transplantation. However, intensified immunosuppression increases the risk of severe infectious complications, particularly in the early post-transplant period. This study investigated whether ABOi-LKT in patients with low baseline anti-ABO isoagglutinin titers can be performed safely without rituximab and thereby reduce infectious complications.

**Methods:**

In this multicenter retrospective cohort study, recipients of ABOi-LKT with low pretransplant anti-ABO titers (≤1:16) were compared according to rituximab use. Clinical outcomes, graft function, rejection episodes, surgical complications, and infectious events were analyzed.

**Results:**

Eleven German transplant centers identified 46 patients who underwent ABOi-LKT without rituximab between 2016 and 2024 and 85 low-titer recipients who received rituximab (single dose 375 mg/m²). Baseline characteristics and median pretransplant anti-ABO titers (1:2) were comparable between groups. Patients underwent a median of two extracorporeal antibody eliminations and received comparable immunosuppression. Graft function at discharge and after 3 and 12 months, as well as proteinuria, did not differ between cohorts. One graft loss due to acute antibody-mediated rejection occurred in the rituximab-free group, whereas two graft losses (one rejection, one BK polyomavirus nephropathy) and one patient death occurred in the rituximab group. Rates of surgical complications (34.8% vs. 36.5%), blood transfusions (21.7% vs. 17.6%), and rejection episodes (15% vs. 20%; p=0.499) were similar. In contrast, infectious complications were significantly more frequent among rituximab-treated patients, with a 2.4-fold increased infection risk (p=0.018). Infection-related hospitalizations occurred significantly more often in the rituximab group (64.6% vs. 31.3%; p=0.003).

**Conclusions:**

ABOi-LKT without rituximab appears safe in recipients with low pretransplant anti-ABO titers. Short-term graft function, graft survival, patient survival, and immunological outcomes were comparable to rituximab-based desensitization. Importantly, omission of rituximab was associated with significantly fewer infectious complications and infection-related hospitalizations, supporting a tailored, lower-intensity immunosuppressive approach in selected low-risk patients.

## Introduction

Kidney transplantation remains the most effective modality for long-term renal replacement therapy compared with established dialysis procedures (1). Owing the persistent shortage of deceased-donor organs, living kidney transplantation continues to play a crucial role in kidney transplantation, with approximately 30% of all living donations being blood group incompatible (2, 3). ABO incompatible living kidney transplantation (ABOi LKT) has been established for many years and provides graft and patient survival rates comparable to ABO-compatible transplantations (4–12). The introduction of effective desensitization protocols by Tydén et al. - combining antibody depletion with CD20-targeted B-cell depletion - has formed the basis of current standard practice in Europe and many other countries (7, 13). Although long-term outcomes are comparable, increasing evidence suggests that early post-transplant complications, particularly infections, occur more frequently, likely attributable to intensified immunosuppression and desensitization therapy (10, 11, 14).

Several studies indicate that reduced doses of rituximab may mitigate the risk of posttransplant infections without compromising efficacy (15–17). Rituximab is a chimeric monoclonal antibody binding to CD20, an antigen expressed on most human B lymphocytes. In the setting of kidney transplantation, it is used for the treatment of recurrent and *de novo* glomerular disease of the transplant, for desensitization of immunized patients prior to kidney transplantation or as part of the desensitization protocol for ABOi LKT. In addition, it is used for the treatment of antibody-mediated rejection (ABMR) or post-transplant lymphoproliferative disorders (18). However, its role in the treatment of ABMR or in desensitization prior to HLA-incompatible transplantation remains questionable (19), although the ESOT ENGAGE consensus considered rituximab as an adjunct for desensitization to prevent antibody rebound and antibody-mediated injury (19, 20). Its necessity has also been questioned in the setting of ABOi LKT as there have been several small, single center experiences demonstrating similar outcomes of ABOi LKT without the use of rituximab in patients with low ABO isoagglutinin titers (21–23). In addition, aside from infusion reactions, rituximab use has been reported to be associated with higher rates of infectious complications (24–26). Especially in the low risk setting of patients with only low levels of ABO isoagglutinin titers, the need for rituximab in desensitization protocols has to be weighed against the risks of its use. Therefore, individualized preconditioning protocols for ABOi LKT based on initial ABO antibody titers are gaining relevance. Several studies tried to investigate, whether ABOi LKT is feasible without either rituximab or antibody removal, or completely avoid any kind of desensitization (23, 27–30). Despite promising data from individual case reports and single center experiences, this approach has not been widely adopted and there are no large trials assessing a rituximab- or desensitization-free protocol. Consequently, in Germany, rituximab remains a central component of the desensitization strategy before ABOi LKT. The aim of this study was to evaluate the experience of German transplant centers with rituximab-free ABOi LKT in a low risk setting of low isoagglutinin titers and assess associated clinical outcomes in a retrospective multi-center approach.

## Materials and methods

In an initial nationwide survey, all German transplant centers performing ABOi LKT were asked to report their desensitization strategies. Seven centers indicated experience in rituximab-free ABOi LKT. Based on this survey, a multicenter retrospective cohort study was conducted, including patients with low ABO isoagglutinin titers, comparing two groups: those who received rituximab pre-transplant (single dose 375mg/m², standard cohort) and those who did not receive rituximab prior to ABOi LKT. In the final analysis 11 German transplant centers contributed all their patients with baseline isoagglutinin titers of ≤ 1:16 having received an ABOi LKT between 2016 and 2024. In this setting four centers used rituximab in all patients, four centers performed ABOi LKT with and without rituximab and three centers performed all low-titer ABOi LKT without the use of rituximab. To detect anti-A and/or anti-B isoagglutinins (IgM and IgG), all centers used the column agglutination technique (Gel Card), with IgG considered relevant for transplantation. Three centers (27.3%) used test erythrocytes, while eight centers (72.7%) performed the measurement using donor erythrocytes. The present analysis was restricted to patients with at least 1 year of follow-up post-transplantation. The rituximab-free cohort comprised 46 patients who did not receive anti-CD20 pre-treatment. The standard cohort included 85 patients with comparable low isoagglutinin titers, all receiving rituximab pre-conditioning. Baseline demographic characteristics (**Table 1**) as well as immunologic parameters (PRA, DSA, anti-ABO titers, desensitization strategy, **Table 2**) and immunosuppressive regimen were recorded.

**Table 1:** Baseline parameters .

Recipients		No Rituximab (n=46)	Standard group (n=85)	p-value
Age, years, mean ± SD		50.6 ± 12.8	47.0 ± 13.5	p = 0.153
Sex, n= (%)	m	32 (69.6%)	54 (63.5%)	p = 0.489
	f	14 (30.4%)	31 (36.5%)	
Height, cm, mean ± SD		175.2 ± 9.9	172.7 ± 11.9	p = 0.296
Weight, kg, mean ± SD		78.0 ± 15.6	77.1 ± 18.4	p = 0.821
CMV-IgG positive, n= (%)		27 (58.7%)	46 (54.1%)	p = 0.616
Diabetes, n= (%)		2 (4.3%)	7 (8.2%)	p = 0.403
Smoking, n= (%)		15 (32.6%)	23 (27.1%)	p = 0.506
Hypertension, n= (%)		45 (97.8%)	78 (91.8%)	p = 0.168
CAD, (y/n), n= (%)		2 (4.3%)	9 (10.6%)	p = 0.221
PAD (y/n), n= (%)		3 (6.5%)	1 (1.2%)	p = 0.091
CVD, (y/n), n= (%)		3 (6.5%)	5 (5.9%)	p = 0.884
No. of Transplants, n= (%)	1	44 (95.7%)	76 (89.4%)	p = 0.214
	2	2 (4.3%)	7 (8.2%)	
	3	0 (0.0%)	2 (2.4%)	
Days on dialysis, median (range)		362 (3-3063)	512 (3-3782)	p = 0.107
Preemptive transplantation, n= (%)		7 (15.2%)	23 (27.1%)	P = 0.135
Donors				
Age, years, mean ± SD		54.2 ± 11.1	54.0 ± 10.8	p = 0.856
Sex, n= (%)	m	16 (34.8%)	33 (38.8%)	p = 0.649
	f	30 (65.2%)	53 (61.1%)	
Height, cm, mean ± SD		169.9 ± 9.4	170.9 ± 8.4	p = 0.475
Weight, kg, mean ± SD		74.2 ± 14.9	75.1 ± 13.2	p = 0.664

SD, Standard Deviation; CMV, Cytomegalovirus; CAD, coronary arterial disease; PAD, peripheral arterial disease; CVD, cerebro vascular disease.

**Table 2:** Immunological characteristics.

Baseline characteristics	No Rituximab (n=46)	Standard group (n=85)	p-value
HLA-A/B/DR Mismatch, mean ± SD	4.0 ± 1.5	3.5 ± 1.5	p = 0.044
median (range)	4 (1-6)	3 (0-6)	
PRA-Level, n= (%)			
<5%	42 (91.3%)	72 (84.7%)	p = 0.538
5-74%	3 (6.5%)	11 (12.9%)	
≥75%	1 (2.2%)	2 (2.4%)	
DSA prior transplant, n= (%)	6 (13.0%)	4 (4.7%)	p = 0.087
Induction, n= (%)			
no induction	0 (0%)	4 (4.7%)	p = 0.077
Basiliximab	46 (100%)	76 (89.4%)	
Thymoglobulin	0 (0%)	5 (5.9%)	
Triple-IS*, n= (%)	46 (100%)	85 (100%)	
Start of IS, days prior transplant, mean ± SD	6.2 ± 4.2	12.9 ± 15.6	p = 0.007
Desensitization, n= (%)			
IVIG	12 (26.1%)	16 (18.8%)	p = 0.375
Rituximab	0 (0%)	85 (100%)	p < 0.001
ABOi-Titer prior treatment, mean ± SD	5.7 ± 4.8	7.9 ± 6.1	p = 0.101
median (range)	4 (0-16)	8 (0-16)	
ABOi-Titer prior transplant, mean ± SD	3.1 ± 3.6	3.3 ± 4.3	p = 0.192
median (range)	2 (0-16)	2 (0-16)	
ABOi-Titer post transplant, mean ± SD	5.3 ± 13.6	1.7 ± 3.0	p = 0.519
median (range)	1 (0-128)	1 (0-16)	
No. of plasma treatments prior transplant,			
mean ± SD	1.8 ± 1.8	2.9 ± 2.6	p = 0.026
median (range)	2 (0-7)	2 (0-14)	

SD, Standard Deviation; PRA, Panel reactive antibody; DSA, Donor specific antibodies; IS, immunosuppression; IVIG, intravenous immunoglobulin, * Triple-IS consists of tacrolimus, mycophenolate and steroids.

### Outcome measures

Outcome and functional parameters included serum creatinine, estimated glomerular filtration rate (eGFR) using CKD-EPI formula, proteinuria, rejection and infection rates as well as surgical complications using the Clavien-Dindo classification. Infectious complications were sub-categorized into urinary tract infection, CMV-viremia, BK Polyomavirus (BKPyV)-viremia, pneumonia or other infections and the need for hospitalization was recorded.

### Statistical analysis

Statistical analysis was performed using Microsoft Excel LTSC MSO (Microsoft Corporation, Redmond, WA, USA), IBM Statistical Package for the Social Sciences (SPSS 31.0,2025, IBM Corporation, NY, USA) and R (Vers. 4.4, 2021, www.R-project.org). Univariate analysis was used to compare baseline characteristics and outcomes, and nonparametric testing for comparison of independent samples from both cohorts. Continuous variables were compared using the Mann–Whitney U-test. Chi-square or Fisher’s exact tests were used for categorical variables as appropriate. The log-rank test was used to compare infection-free survival between the cohorts. According to common conventions, the statistical significance level was set at p <0.05. The study was reviewed and approved by the Aachen University Hospital Ethics Committee (CTC-A-Ref. Nr. 22-203).

## Results

131 patients with a baseline anti-ABO titer of ≤ 1:16 were identified in eleven German transplant centers. 46 of them received ABOi LKT using a rituximab-free regimen, and 85 patients using pretransplant rituximab in standard dose. Baseline characteristics such as age, sex, time on dialysis as well as comorbidities did not differ between the different cohorts (**Table 1**). In addition, donors also were comparable between the cohorts (**Table 1**). Follow-up was one year.

### Immunology and immunosuppression

In terms of immunology, the rituximab-free cohort showed significantly poorer HLA-matching with a median HLA-A/B/DR mismatch of 4 (range 1-6) compared to a median mismatch of 3 in the rituximab cohort (range 0-6, p=0.044). 13% of patients in the rituximab-free group vs. 5% in the group with rituximab had DSA prior to transplantation. This difference did not reach statistical significance (**Table 2**). Immunosuppression in both cohorts was comparable using a triple combination of tacrolimus, mycophenolate and steroids. All patients in the rituximab-free cohort received basiliximab induction. In the rituximab cohort 6% received anti thymocyte globulin (ATG), 89% basiliximab and 5% of patients did not receive any induction therapy. Approximately 20% of patients in either cohort received IVIG as part of their desensitization protocol. Overall, there was no statistically significant difference regarding the type of immunosuppression between the two groups (p=0.077). However, immunosuppression was initiated significantly earlier in the rituximab group (12.9 vs. 6.2 days prior to transplant, p=0.007).

**Figure 1:**
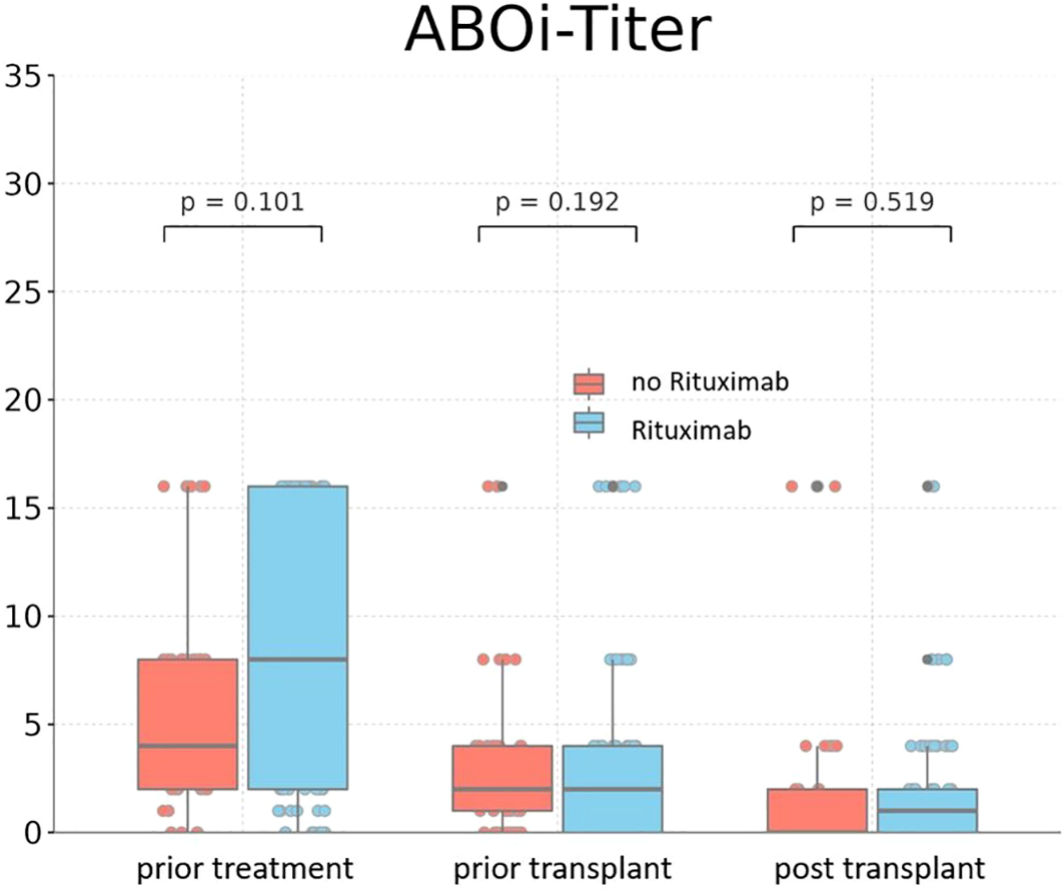
Overview of anti-ABO titers between both groups prior treatment, prior and post transplant.

ABO isoagglutinin titers at baseline and immediately before transplantation did not differ between the two cohorts (**Figure 1**). Median ABO isoagglutinin titer prior to therapy was 1:4 in patients transplanted without rituximab and 1:8 in patients with rituximab. However, despite comparable isoagglutinin titers, significantly more extracorporal antibody eliminations were performed in the rituximab cohort (1.8 vs. 2.9; p=0.026). Immunological characteristics are shown in **Table 2**. Post-transplant antibody rebound requiring additional treatment occurred in one patient (2.2%) in the rituximab-free group and in no patient in the rituximab group. Seven patients in the rituximab group underwent postoperative antibody elimination according to center-specific protocols despite antibody titers remaining ≤1:16.

### Graft and patient outcome

**Figure 2:**
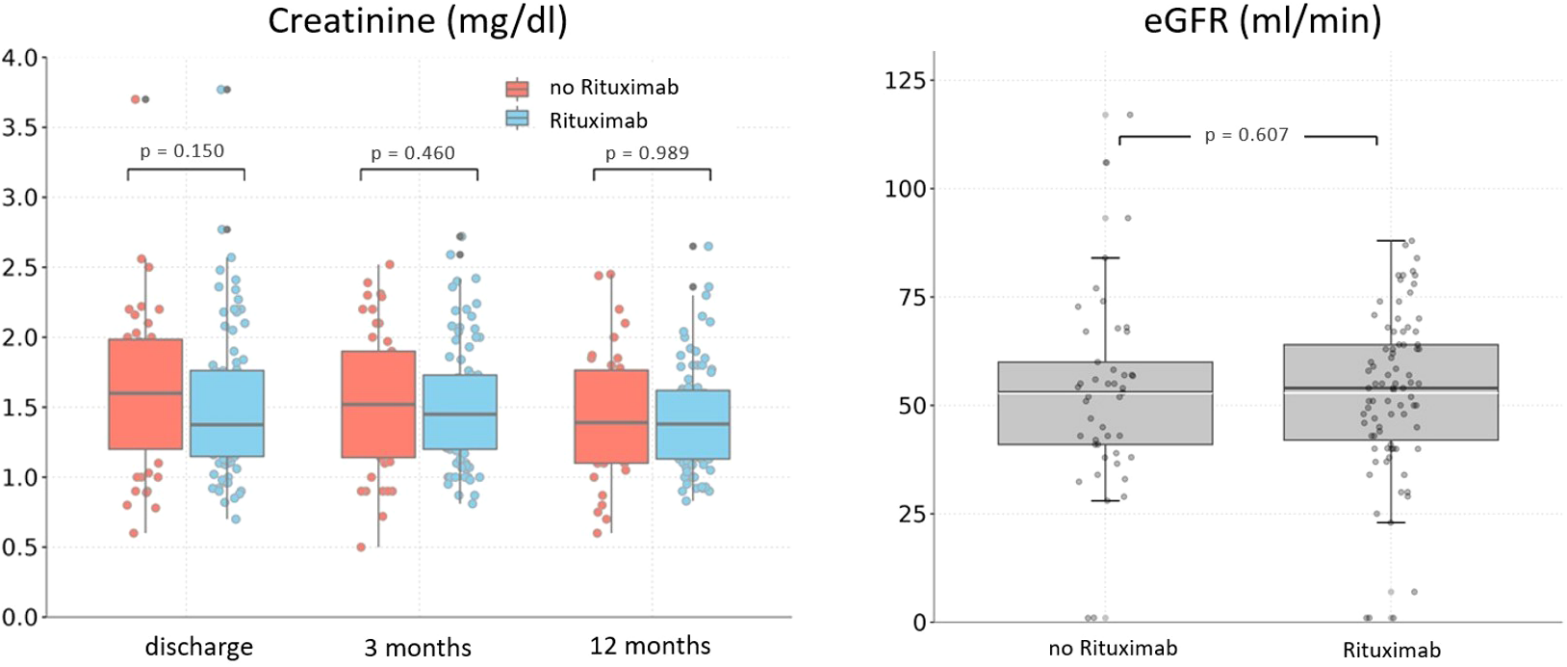
Creatinine at discharge, 3 months and 12 months post transplant, eGFR at 12 months post transplant.

Renal function at discharge, after 3 and 12 months did not differ between both cohorts (**Figure 2**). Mean discharge serum creatinine in the rituximab-free cohort was 1.7 ± 0.8 mg/dl as compared to 1.5 ± 0.6 mg/dl in the standard cohort (difference not statistically significant, **Table 3**). Graft function at 1 year was likewise similar between both cohorts. Proteinuria was generally low with a median at 71 mg/g in the cohort without rituximab vs. 87 mg/g in the standard cohort (p=0.493). In both cohorts, graft loss occurred in 2% of ABOi LKT within the first year. In the rituximab-free cohort, there was one immunological graft loss due to acute humoral rejection with need for graft nephrectomy on day four. This particular blood group A recipient showed an initial ABO isoagglutinin titers of 1:16 against the AB-donor and received a session of four unspecific immunoadsorption’s followed by IVIG. Post transplantation, primary dysfunction occurred. Biopsy confirmed acute humoral rejection; however, neither DSA nor HLA/non-HLA antibodies were detected. Genetic testing for atypical hemolytic syndrome was unremarkable. In patients receiving rituximab, two patients lost their graft within the first year. One patient with blood group A (ATG-induction, anti-B 1:8, five times specific immunoadsorption prior to transplant and pre-op titer of 1:1), developed acute graft loss on day one posttransplant. In this patient intraoperative graft perfusion was already impaired. Histological workup demonstrated renal cortical necrosis and infarction, probably due to acute rejection. However, HLA-antibodies were not detectable and ABO isoagglutinin titer was measured as pretransplant. A second graft loss was associated with BKPyV-viremia and biopsy proven BKPyV-nephropathy 11 months posttransplant. In the standard cohort, a patient with prune-belly-syndrome and severe abdominal adhesions resulting from a previous transplant and the underlying disease, died after diffuse intrabdominal bleeding with hemorrhagic shock along with local peritonitis associated with a fistula of the ascending colon. There were no deaths in the cohort without rituximab.

**Table 3:** 1-year outcome kidney function.

Baseline characteristics		No Rituximab (n=46)	Standard group (n=85)	p-value
Primary function (y/n), n= (%)	No	3 (6.5%)	3 (3.5%)	p = 0.436
	yes	43 (93.5%)	82 (96.5%)	
Creatinine at discharge, mg/dl mean ± SD		1.7 ± 0.8	1.5 ± 0.6	p = 0.150
Creatinine at 3 months, mg/dl mean ± SD		1.6 ± 0.5	1.5 ± 0.4	p = 0.460
Creatinine at 12 months, mg/dl mean ± SD		1.4 ± 0.5	1.5 ± 0.7	p = 0.989
eGFR at 12 months, ml/min mean ± SD		55.2 ± 19.4	54.8 ± 16.1	p = 0.607
PCR at 12 months, mg/g median (range)		71 (0-778)	87 (0-3570)	p = 0.493

SD, Standard Deviation; eGFR, estimated Glomerular filtration rate (ml/min); PCR, Protein/Creatinine-Ratio.

### Treatment associated complications

**Figure 3:**
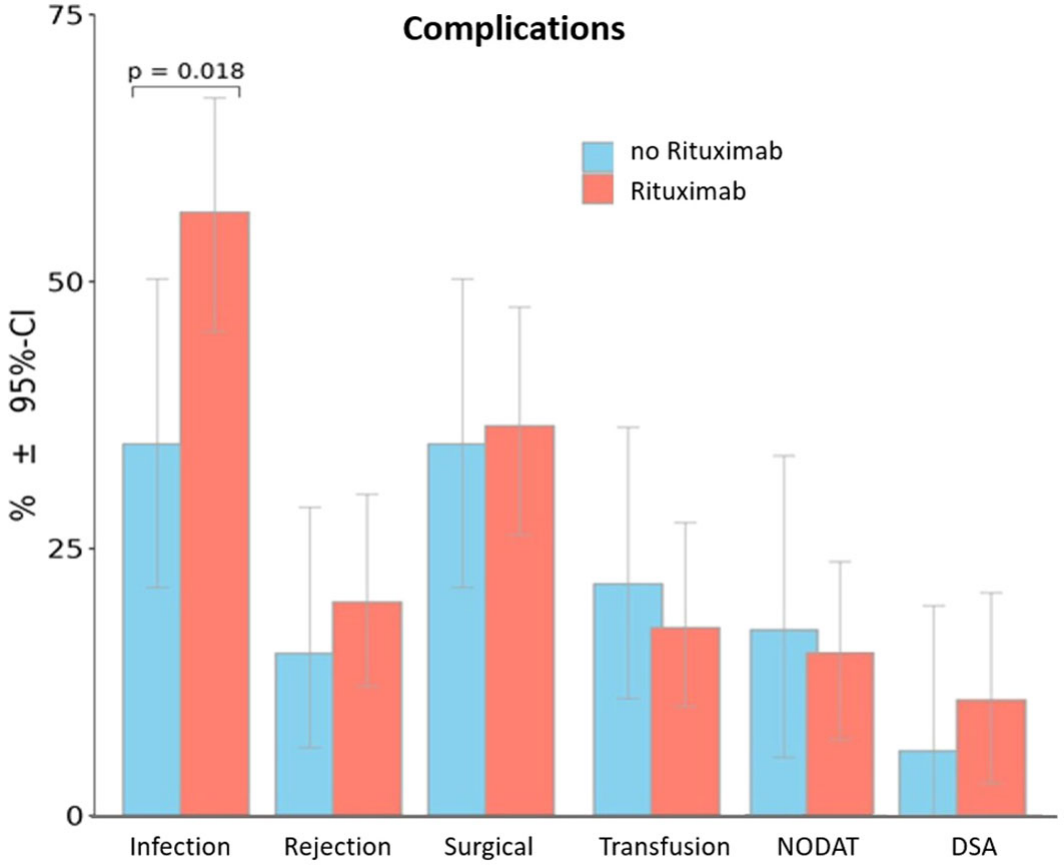
Overview of complications between both groups 12 months post transplant.

Overall, treatment associated complications were similar between the two cohorts (**Figure 3**; **Table 4**).

**Table 4:** 1-year outcome complications.

Baseline characteristics		No Rituximab (n=46)	Standard group (n=85)	p-value
DSA, n= (%)		3 (6.5%)	10 (11.8%)	p = 0.340
Infections, n= (%)		16 (34.8%)	48 (56.5%)	p = 0.018 OR = 2.432 [1.157; 5.113]
Rejections, n= (%)		7 (15.2%)	17 (20.0%)	p = 0.499 OR = 1.393 [0.531; 3.653]
Borderline		3 (6.5%)	5 (5.9%)	
TCMR		2 (4.3%)	8 (9.4%)	
ABMR		2 (4.3%)	4 (4.7%)	
Blood Transfusions, n= (%)		10 (21.7%)	15 (17.6%)	p = 0.569 OR = 0.771 [0.315; 1.889]
Surgical complications, n= (%)		16 (34.8%)	31 (36.5%)	p = 0.848 OR = 1.076 [0.508; 2.280
Clavien-Dindo	I	4 (25.0%)	8 (25.8%)	p = 0.952
	II	1 (6.3%)	3 (9.7%)	
	III	11 (68.8%)	20 (64.5%)	
NODAT, n= (%)		8 (17.4%)	13 (15.3%)	p = 0.756

DSA, Donor specific antibodies; NODAT, New onset diabetes after transplantation; TCMR, T-Cell mediated rejection; ABMR, Antibody mediated rejection.

Surgical complications were observed in about 35% of all patients, whether treated with rituximab or not. Between groups, the Clavien-Dindo classification of these complications showed no significant differences (**Table 4**). Blood transfusion rates were comparable between both cohorts (21.7% vs. 17.6%, p= 0.596). Miscellaneous complications such as inguinal hernia, depression, aHUS, recurrence of original kidney disease etc. were not different between the cohorts.

### Infections

**Figure 4:**
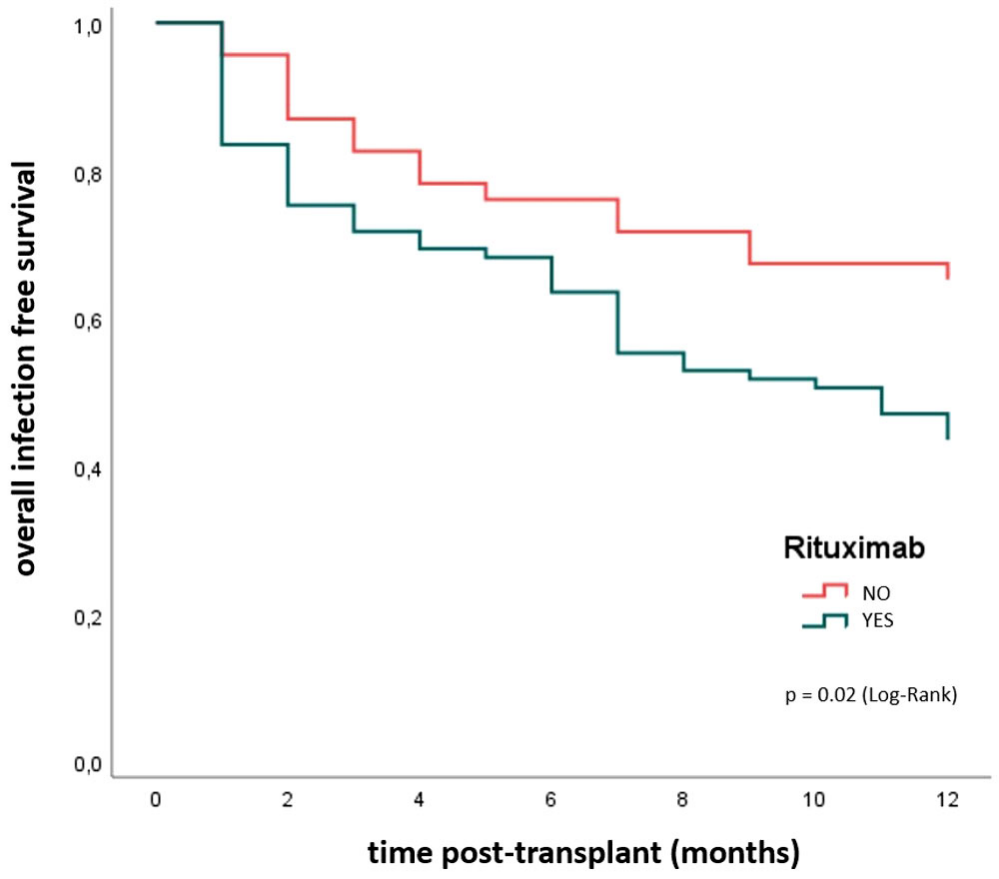
Kaplan-Meier curve for infection-free survival.

Infectious complications during the first year post transplantation occurred more frequently in patients receiving rituximab than in those without (56.5% vs. 34.8%; OR 2.43, 95% CI 1.16–5.11; p=0.018). Additionally, infections requiring hospitalization were markedly more common when rituximab was given (36.5% vs. 10.9%; OR 4.70, 95% CI 1.69–13.18; p = 0.003), whereas ambulatory infections did not differ significantly between groups. The timing of infections is depicted in Kaplan–Meier curves for overall infection-free survival during the first 12 months after kidney transplantation, stratified by Rituximab exposure (**Figure 4**). Patients who did not receive Rituximab demonstrated a consistently higher infection-free survival compared to those treated with Rituximab. Separation of the curves occurs early after transplantation and persists throughout follow-up. Apart from a significantly higher number of urinary tract infections when rituximab was administered, the remaining distribution of infection categories did not significantly differ between both cohorts (**Table 5**).

**Table 5:** Infectious complications and categories Among All Patients.

Outcome	No Rituximab (n=46)	Standard group (n=85)	p-value
Overall Infections, n= (%)	16 (34.8%)	48 (56.5%)	p = 0.018 OR = 2.43 [1.16; 5.11]
Infections with hospitalization, n= (%)	5 (10.9%)	31 (36.5%)	p = 0.003 OR = 4.70 [1.69; 13.18]
CMV-viremia, n= (%)	1 (2.2%)	6 (7.1%)	p = 0.420
BKPyV-viremia, n= (%)	6 (13.0%)	11 (12.9%)	p = 1.00
UTI, n= (%)	1 (2.2%)	17 (20.0%)	p = 0.003
Pneumonia, n= (%)	4 (8.7%)	4 (4.7%)	p = 0.450
Others infections, n= (%)	4 (8.7%)	10 (11.8%)	p = 0.769

CMV, Cytomegalovirus; BKPyV, BK Polyomavirus; UTI, Urinary tract infection.

### Rejections and DSA

Overall, the rejection rate after one year was comparable in both cohorts (**Table 4**). Notably, the rituximab-free cohort showed a trend towards fewer rejections (15% vs. 20%, p=0.5) and less donor-specific HLA antibodies (6.5% vs. 11.8%, p=0.34) without statistical significance. This occurred despite higher HLA-mismatches, more preformed DSA prior to transplant, a significantly earlier start of immunosuppression and more extracorporeal antibody eliminations in the rituximab cohort (**Table 2**). Similar to the overall incidence of rejections, the number of antibody-mediated rejections was comparable in both cohorts (4% in the cohort without rituximab and 5% in the cohort with rituximab, **Table 4**). As written above, there was one immunological graft loss in each group.

## Discussion

This retrospective multicenter cohort study assessed the efficacy and safety of ABOi LKT using preoperative desensitization protocols that include rituximab compared with protocols that omit rituximab in a low immunological risk setting. The cohorts comprised all patients from 11 German transplant centers who underwent ABOi LKT with initial ABO isoagglutinin titers ≤1:16 between 2016 and 2024. These centers - representing approximately one third of all German kidney transplant programs - including small and medium-sized units, thereby providing a representative cross-section of ABOi LKT practice in Germany.

In this cohort, we demonstrate that ABOi LKT without rituximab in patients with low baseline isoagglutinin titers seems to be safe, yielding comparable one-year outcomes regarding graft function, graft survival, and patient survival. Likewise, rates of surgical complications, blood transfusions, and acute rejections did not differ between groups while infections were less frequently in the cohort without rituximab.

This study corroborates findings from previous single-center investigations by demonstrating, in a multicenter German cohort, that patients undergoing ABOi LKT with perioperative antibody removal and standard immunosuppression—but without rituximab—can achieve short- to medium-term outcomes comparable to those receiving more intensive desensitization regimens (15, 23, 30, 31). Notably, we did not see increased rates of antibody mediated rejection in our cohort without rituximab. Insufficient depletion of ABO isoagglutinin antibodies in the setting of ABOi LKT can lead to hyperacute rejection or, in a less severe form, to ABMR. However, with modern desensitization protocols that include rituximab and antibody removal, early ABMR has become rare (31). It could therefore be hypothesized that omitting rituximab from the desensitization protocol in ABOi LKT would increase the risk of ABMR and immunological graft loss. Nevertheless, most retrospective cohort studies have not shown a higher incidence of ABMR when rituximab-free protocols were used in low titer situations (17, 21, 23).

In contrast, a single-center retrospective analysis from Australia reported an 8.8-fold higher odds ratio for acute rejection in ABOi LKT without rituximab compared to a historical cohort treated with rituximab. In addition to a generally higher incidence of rejection, there was also an increased number of ABMR cases (32). One possible explanation for this finding is that 40% of patients in the rituximab-free cohort had donor-specific antibodies, compared to only 22% in the rituximab-treated cohort and a strong association between anti-ABO titer rebound and ABMR in the rituximab-free group.

In our study, the incidence of rejection was similar between cohorts (15% without rituximab versus 20% with rituximab), with two cases of ABMR (4%) in the cohort without rituximab and four cases (5%) in the standard treatment cohort.

Ashimine et al. assessed the incidence of *de novo* HLA antibodies in patients after ABO-compatible and ABO-incompatible living kidney transplantation. They found no difference in the incidence of ABMR between the cohorts, nor in the occurrence of *de novo* HLA antibodies (33). Interestingly, in our multicenter study, a higher proportion of patients in the cohort without rituximab had DSA before transplantation (13% vs. 4,7%), whereas fewer patients in this cohort had DSA one year after transplantation (6.5% vs. 11.8%). Both studies suggest, that there is no strong effect of rituximab on DSA development after transplantation.

Aside from similar efficacy with and without rituximab with respect to graft function, graft and patient outcome, we saw a reduced rate of infections in the cohort that did not receive rituximab. It could be hypothesized that this was attributable to the overall lower immunosuppressive burden in this cohort. The impact of intensified immunosuppression in ABOi LKT compared with ABO-compatible transplantation was assessed by Schachtner et al. They measured alloreactive T-cell responses, lymphocyte subpopulations, and cytokine profiles in patients undergoing ABOi- and ABOc-LKT and found that despite comparable transplant outcomes, ABOi LKT recipients exhibited higher rates of CMV infection, BKPyV-nephropathy and severe sepsis. Immunological analyses demonstrated impaired CMV- and BKV-specific T-cell immunity and reduced frequencies of alloreactive T cells in the ABOi cohort. The authors postulated that rituximab-mediated B-cell depletion—affecting antigen-presenting cell function—may attenuate T-cell activation and thereby compromise infection control in ABOi LKT recipients (34, 35). In our study, we could clearly see fewer overall infections and less severe infections requiring hospitalization without rituximab desensitization. Due low numbers, we could not see a difference between the different cohorts with respect to CMV- or BKPyV-viremia. In this context, the higher rate of infections within the rituximab group maybe associated with the higher number of extracorporeal antibody elimination sessions (mean 1.8 ± 1.8 vs. 2.9 ± 2.6, p=0.026). The intensity of apheresis procedures seems to be associated with increased infection risk (36).

Another important question is whether the dose of rituximab itself contributes to the increased risk of infectious complications. Lee et al. addressed this in a meta-analysis of clinical studies including 4,256 AB0i-LKT recipients treated with different rituximab doses (200 mg, 200–500 mg, or ≥500 mg) as part of their desensitization protocols (37). While patient survival, graft failure, and overall bacterial and viral infection rates were comparable across dosing groups, higher rituximab doses were associated with a significantly increased risk of infection-related mortality compared with the 200 mg dose.

Similarly, Thukral et al. conducted a prospective study evaluating even lower rituximab doses (100 mg vs. 200 mg) in AB0i-LKT recipients (38). Although both groups demonstrated comparable patient and graft survival, graft function, and rejection rates, infectious complications occurred more frequently in the 200 mg group. These findings support the notion that even relatively low rituximab doses (200mg) may contribute to an elevated risk of infection.

In contrast, Okada et al. compared two protocols in ABOi LKT recipients with low baseline isoagglutinin titers—one without rituximab and one with 100 mg low-dose rituximab (39). Low-dose rituximab was associated with a significantly reduced incidence of antibody-mediated rejection and lower post-transplant isoagglutinin titers, while adverse events, including infections, did not differ between groups. These findings suggest that very low-dose rituximab (100mg) may offer immunologic benefit without increasing risk for infections. In our cohort, however, we did not observe higher rejection rates in patients who did not receive rituximab compared to a single dose group of 375mg/m². Also, post-transplant isoagglutinin titers were comparable between both groups.

In conclusion, our multicenter retrospective cohort study provides real-world data in ABO-incompatible living kidney transplantation in patients with low isoagglutinin titers of ≤ 1:16, with detailed immunological characterization and comprehensive assessment of graft and patient outcomes, including rejection rates, donor-specific antibodies, and risk for infections. The inclusion of eleven German transplant centers, comprising one third of all German transplant centers, enhances the generalizability of the findings. A limitation of this retrospective multicenter study is the potential for selection bias. Four centers performed ABOi LKT with and without rituximab. In these centers, the decision to administer rituximab in patients with low baseline isoagglutinin titers was made at the discretion of the treating physician rather than according to a predefined protocol. To reduce potential selection bias, all consecutive eligible ABO-incompatible living kidney transplant recipients with baseline isoagglutinin titers ≤1:16 treated between 2016 and 2024 were included, without further selection. Baseline demographic and immunological characteristics were comparable between groups (**Tables 1** and **2**), but unmeasured confounders cannot be excluded.

While the retrospective non-randomized design and modest sample size limits statistical power, this multicenter analysis provides a representative real-world dataset on a practice that remains underrepresented in the literature. It confirms similar results in respect of kidney function as well as graft and patient survival in the cohort that was transplanted without the use of rituximab. In addition to similar rate of complications, the omission of rituximab might be associated with a reduced risk of severe infections, corroborating earlier single-center findings. These findings contribute to current efforts to individualize immunosuppressive regimens, improve patient safety, and refine risk-adapted treatment strategies in kidney transplantation.

## Data Availability

The raw data supporting the conclusions of this article will be made available by the authors, without undue reservation.
